# Functional analysis of a triplet deletion in the gene encoding the sodium glucose transporter 3, a potential risk factor for ADHD

**DOI:** 10.1371/journal.pone.0205109

**Published:** 2018-10-04

**Authors:** Nadine Schäfer, Maximilian Friedrich, Morten Egevang Jørgensen, Sina Kollert, Hermann Koepsell, Erhard Wischmeyer, Klaus-Peter Lesch, Dietmar Geiger, Frank Döring

**Affiliations:** 1 Department of Molecular Plant Physiology and Biophysics, Julius-von-Sachs-Institute, University of Würzburg, Würzburg, Germany; 2 Division of Molecular Psychiatry, Center of Mental Health, University Hospital of Würzburg, Würzburg, Germany; 3 Division of Molecular Electrophysiology, Institute of Physiology, University of Würzburg, Würzburg, Germany; 4 Laboratory of Psychiatric Neurobiology, Institute of Molecular Medicine, Sechenov First Moscow State Medical University, Moscow, Russia; 5 Department of Psychiatry, Psychosomatics and Psychotherapy, Center of Mental Health,University Hospital of Würzburg, Würzburg, Germany; 6 Department of Neuroscience, School for Mental Health and Neuroscience (MHeNS), Maastricht University, Maastricht, The Netherlands; Universidad de la Laguna, SPAIN

## Abstract

Sodium-glucose transporters (SGLT) belong to the solute carrier 5 family, which is characterized by sodium dependent transport of sugars and other solutes. In contrast, the human SGLT3 (hSGLT3) isoform, encoded by *SLC5A4*, acts as a glucose sensor that does not transport sugar but induces membrane depolarization by Na^+^ currents upon ligand binding. Whole-exome sequencing (WES) of several extended pedigrees with high density of attention-deficit/hyperactivity disorder (ADHD) identified a triplet ATG deletion in *SLC5A4* leading to a single amino acid loss (ΔM500) in the hSGLT3 protein imperfectly co-segregating with the clinical phenotype of ADHD. Since mutations in homologous domains of hSGLT1 and hSGLT2 were found to affect intestinal and renal function, respectively, we analyzed the functional properties of hSGLT3[wt] and [ΔM500] by voltage clamp and current clamp recordings from cRNA-injected *Xenopus laevis* oocytes.

The cation conductance of hSGLT3[wt] was activated by application of glucose or the specific agonist 1-desoxynojirimycin (DNJ) as revealed by inward currents in the voltage clamp configuration and cell depolarization in the current clamp mode. Almost no currents and changes in membrane potential were observed when glucose or DNJ were applied to hSGLT3[ΔM500]-injected oocytes, demonstrating a loss of function by this amino acid deletion in hSGLT3. To monitor membrane targeting of wt and mutant hSGLT3, fusion constructs with YFP were generated, heterologously expressed in *Xenopus laevis* oocytes and analyzed for membrane fluorescence by confocal microscopy. In comparison to hSGLT3[wt] the fluorescent signal of mutant [ΔM500] was reduced by 43% indicating that the mutant phenotype might mainly result from inaccurate membrane targeting. As revealed by homology modeling, residue M500 is located in TM11 suggesting that in addition to the core structure (TM1-TM10) of the transporter, the surrounding TMs are equally crucial for transport/sensor function.

In conclusion, our findings indicate that the deletion [ΔM500] in hSGLT3 inhibits membrane targeting and thus largely disrupts glucose-induced sodium conductance, which may, in interaction with other ADHD risk-related gene variants, influence the risk for ADHD in deletion carriers.

## Introduction

Membrane transport of glucose in mammalian cells is mediated either by members of the SLC2 or the SLC5 transporter family. Glucose transporters [GLUTs] of the SLC2 family facilitate diffusion of D-glucose across the plasma membrane [1]. In contrast, members of the SLC5 family (sodium-glucose transporters or symporters; SGLTs) mediate co-transport of D-glucose in expense of the electrochemical sodium gradient across the plasma membrane. The secondary active transport by SGLTs allows the accumulation of D-glucose in various cell types [2]. Genes of SGLTs code for membrane proteins that consist of 14 transmembrane segments with both amino- and carboxy-terminus located on the extracellular side as revealed by site-directed mutagenesis and crystal structure analysis (reviewed by [3]). Studies with human SGLT1 (hSGLT1) and the model homologue from *Vibrio parahaemolyticus* (vSGLT) have shown that fully functional transporters are formed by monomeric proteins [4, 5]. X-ray analysis of crystals from vSGLT discovered a core structure of inverted repeat topology of TM1-TM5 and TM6-TM10 with no amino acid homology but structural domains that can be superimposed [6]. The core structure is flanked by a single transmembrane segment on the amino terminal side and three of them at the carboxy terminus, named -TM1 and TM11-13, respectively (according to [7]). Within this core structure (TM1–TM10) specific amino acids were identified that are essential either for substrate or sodium binding. Moreover, mechanisms for the transfer of ions and substrate molecules from the extracellular to the intracellular side are attributed to this core element of the protein [6]. In contrast, the function and relevance of the additional TM segments (-TM1 and TM11-13) on the N- and C-termini are poorly understood. Evidence for the importance of several specific amino acids in hSGLTs was deduced from intestinal and renal diseases caused by mutations in these transporters [8, 9, 10].

In the intestinal tract the high affinity transporter hSGLT1 is responsible for the uptake of glucose and galactose across the apical membrane into intestinal cells. Mutations in the hSGLT1 lead to glucose- galactose malabsorption (GGM, [11]). The majority of mutations are missense, distributed throughout the protein and most commonly causes reduced Na^+^/glucose transport activity by reduced glucose affinity or improper membrane targeting of the protein [8]. Human SGLT2 is expressed in the kidney and retrieves most of the glucose from the proximal tubule by coupling one sodium to the transport of one glucose molecule. In the distal part of the proximal tubule more potent reabsorption of glucose is coupled to two sodium ions by hSGLT1. Familial renal glucosuria (FRG) is a rare autosomal recessive disorder where glucose is excreted in the urine, when blood glucose level is normal. Missense, deletion and nonsense mutations in the coding region of hSGLT2 were identified in patients with FRG of different severity [9, 10]. Attempts to link specific mutations to the severity of glucosuria failed due to ineffective expression of hSGLT2 in heterologous systems. Instead hSGLT3 [*SLC5A4*), was efficiently inserted into the plasma membrane when heterologously expressed in *Xenopus laevis* oocytes, but it was unable to transport glucose. However, under these conditions glucose caused a Na^+^-dependent depolarization of the cell, which was reversed by the SGLT-specific antagonist phlorizin [12]. Thus, based on its electrophysiological properties hSGLT3 is accepted to be a glucose sensor and not Na^+^/glucose transporter. High expression of hSGLT3 was found in small intestine and skeletal muscle [12, 13] whereas in other tissues including brain, hSGLT3 appears to be expressed at a lower level (reviewed by [14, 15]).

Attention-deficit/hyperactivity disorder (ADHD) is a common neurodevelopmental disorder characterized by increased motor activity, a persistent pattern of inattention and impulsivity that interferes with academic functioning, increased risk of drug abuse and negative consequences for family and peer relations [16]. Although environmental influences (such as low birth weight, childbirth complications, toxin exposure and food additives) have been identified, genetic factors are considered as the critical component of ADHD [17]. Recently, several single-nucleotide polymorphisms and duplication of SLC2A3 encoding the neuronal glucose transporter-3 (GLUT3) were found to be associated with ADHD. Upon testing, patients display deficits in working memory and cognitive response control, suggesting altered genes of the glucose metabolism as chancy for the development of ADHD. [18].

Using whole-exome sequencing of several multi-generational pedigrees of German descent with high density of ADHD we identified a triplet ATG deletion in *SLC5A4* leading to a loss of the amino acid methionine in the 11^th^ transmembrane segment (TM11) in hSGLT3 (ΔM500) co-segregating—although imperfectly—with the clinical phenotype of ADHD. Here, we analyzed functional properties of the SGLT3[ΔM500] mutation in the *Xenopus laevis* oocyte expression system. Alterations in membrane targeting and electrophysiological properties indicate that TM11 located outside the core structure of hSGLT3, is essential for the mechanism of ion transport and glucose sensing.

## Material and methods

### Whole-exome sequencing and data analysis

To identify rare and disruptive genetic variants associated with ADHD whole-exome sequencing (WES) was conducted in several multi-generational families of German descent (citizens in at least third generation) with persistent ADHD. Diagnostic procedure for family members according to DSM-IV criteria has been described previously [19]. The study was approved by the Ethics Committee of the Julius-Maximilians-University of Würzburg. Written informed consent was obtained from all participating individuals.

For each family, two or more affected family members were strategically selected for WES based on meiotic distance and/or position in the pedigree. The exome was targeted by Agilent Sure Select Human All Exon 50Mb Target Enrichment kit (Agilent Technologies, Santa Clara, CA) and sequenced by single-end sequencing on the 5500xl SOLiD System (Life Technologies, Carlsbad, CA) as previously described [20]. High quality reads were mapped to the hg19 reference genome (UCSC genome browser) using the Lifescope 2.1 software (http://www.lifetechnologies.com/lifescope/) with default parameters. In addition, the SOLiD Lifescope Software v2.1 was used to call single nucleotide variants (SNVs) using the diBayes algorithm. Variant annotation was done by M. Klein and B. Franke at the Department of Human Genetics of the Radboud University Medical Center using a pipeline developed in-house [21].

### Segregation analysis

**Fig 1:**
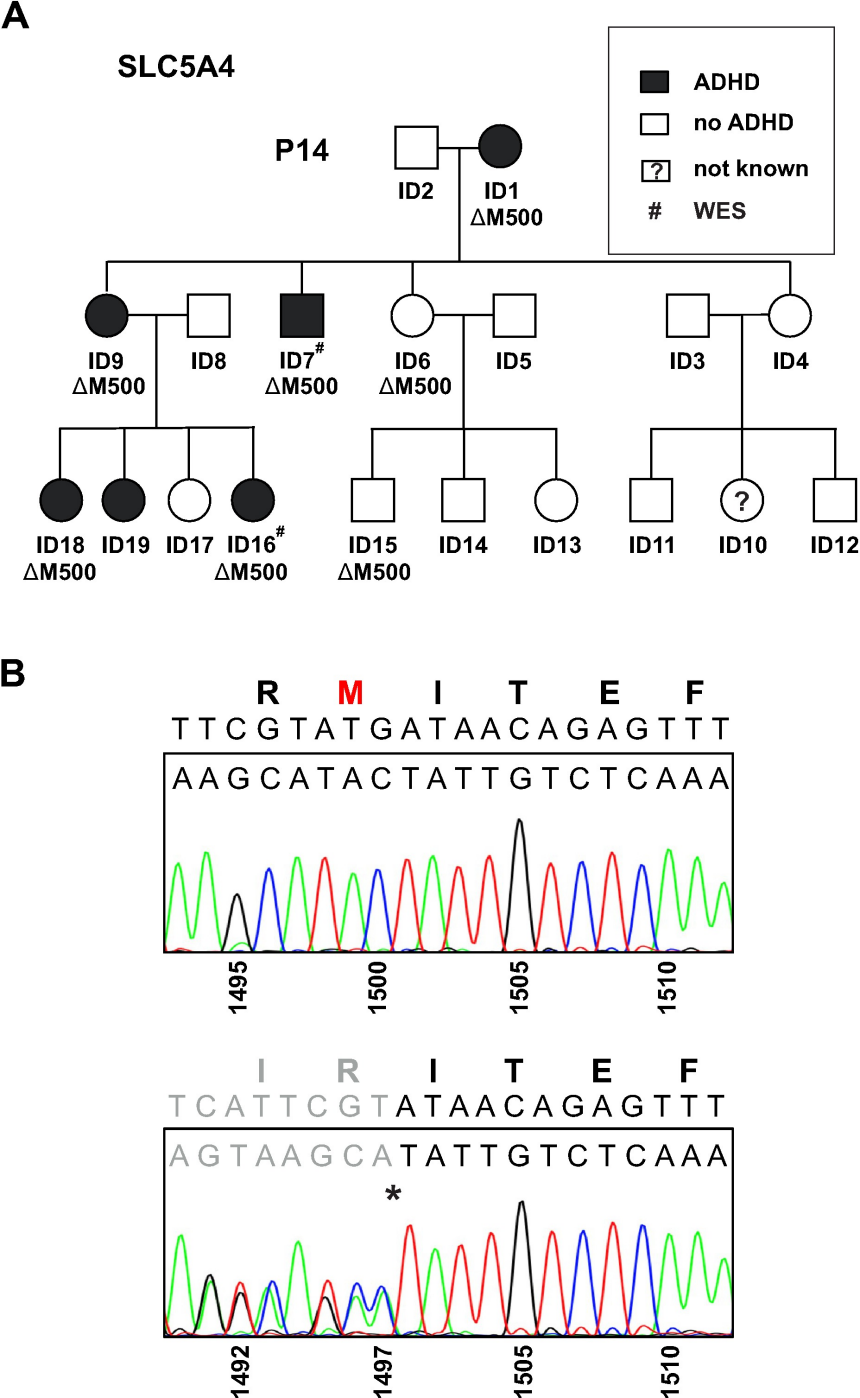
WES screening identified a mutation in the glucose sensor SGLT3 in ADHD patients. (A) Pedigree of family P14 with a heterozygous triplet ATG deletion in *SLC5A4* leading to a single amino acid loss in SGLT3 (ΔM500), imperfectly co-segregating with the clinical phenotype of ADHD. Shading of squares (male) and circles (female) indicate affected (black) and healthy (white) family members with the *SLCLC5A4* genotype carriers indicated below. (B) Representative Sanger sequencing of homozygous (upper panel) and heterozygous (lower panel) genotypes demonstrate a deletion of three nucleotides (c.1498-1500delATG) in heterozygous individuals (asterisk) resulting in a deletion of methionine 500 in the SGLT3 protein. Numbering refers to nucleotides of the open reading frame derived from the healthy (upper panel) and the mutated (lower panel) alleles.

To validate the presence of selected rare variants in the individuals subjected to WES and to allow segregation analysis, all individuals of family P14 illustrated in a pedigree (Fig 1A), were genotyped for the triplet ATG deletion (c.1498-1500delATG) using PCR-based DNA sequencing (Fig 1B). The locus of interest was amplified by conventional PCR and sequenced by direct Sanger sequencing (details and primer sequences are available upon request). Genotypes obtained for the variant were used to analyze the segregation with categorical diagnosis of ADHD.

### Conventional RT-PCR (endpoint-PCR)

To verify the expression profile of hSGLT3 1μg total RNA from different human brain tissues (BD Biosciences, Erembodegem, Belgium) was reverse transcribed in a final volume of 20 μl using iScript cDNA Synthesis Kit (BioRad Laboratories, Hercules, CA, USA). Gene specific and intron-spanning primers (forward 5’-gggacaacttgacaatcagtgcc-3’; reverse 5’-gccaacatcaacgccacagtg-3’) were generated to selectively amplify a 348 base pair fragment of hSGLT3 cDNA. Using 1 μl cDNA and hot-start Taq Polymerase (Qiagen) PCRs were run on a standard thermo cycler (model T3; Biometra, Göttingen, Germany) with the following conditions (total volume 20 μl): initial denaturation step 5 min 94°C; 32 cycles: 45 s 94°C, 45 s 55,5°C, 45 s 72°C; final elongation step 3 min. 72°C. Complete PCR samples were analyzed on a 4% agarose gel.

### Quantitative real-time RT-PCR

In order to quantify the relative expression of hSGLT3 in diverse human brain tissues we apply two-step quantitative real-time RT-PCR (qRT-PCR). Total RNA (0.5 μg) of each tissue was reverse transcribed with random hexamer and oligo-dT-primers using iScript cDNA Synthesis Kit (BioRad Laboratories, Hercules, CA, USA). Reaction mixtures were supplemented with ddH_2_O to a final volume of 100μl. Real-time PCR and data analysis were performed in a total volume of 25 μl using 96-well plates and an iCycler IQ system (BioRad Laboratory). One microliter cDNA, 12.5 μl QuantiTect SYBRGreen PCR Kit (Qiagen), 2.5 μl QuantiTect Primer Assay (Qiagen) and 10 μl ddH_2_O were subjected to each microwell. All PCRs were run in triplicate and conditions were the same as applied in endpoint-PCR (see above). Amplicons were quantified with the comparative threshold cycle (C_T_) method computed by iCyler IQ software (BioRad Laboratories). To normalize these qRT-PCR data the expression of two housekeeping genes (hGAPDH, hUBC) were analyzed in cDNA samples of different tissues Using gNorm algorithm developed by Vandesompele et al. [22] the stability of each housekeeping gene was tested and a normalization factor for each cDNA sample was calculated. Data were analysed with Excel software (Microsoft Corporation) and presented as mean ± SE.

### Molecular cloning and *in vitro* transcription

The cDNAs coding for hSGLT1 (*SLC5A1*, 11]) and hSGLT3 (*SLC5A4*; [12]) were cloned into pGEM-derived USER-compatible oocyte expression vectors using an advanced uracil-excision-based cloning technique as described by Nour-Eldin et al. [23]. For localization of hSGLT3, hSGLT1 and their mutants ΔM500 and ΔI501 corresponding cDNAs were fused to yellow fluorescence protein (YFP) at the C-terminal end. Fusion constructs were engineered by subcloning cDNAs into a YFP oocyte expression vector pNB1uYFP [23]. To introduce site-specific mutations QuikChange mutagenesis kit from Aligent Technologies (Santa Clara, CA) was used according to the manufacturer’s instructions. Nucleotide sequences of engineered constructs were checked by sequencing service (Eurofins MWG Operon, München, Germany) and ApE analysis software (edited by M. Wayn Davis, UT). m7G(59)G-capped sense cRNAs were synthesized using T7 RNA-polymerase. cRNAs were prepared *in vitro* using the the Ampli Cap-Max^TM^ T7 High Yield Message Maker Epicentre Kits (Biozym, Hessisch Oldendorf, Germany) according to the manufacturer’s specifications.

### Oocyte preparation and heterologous expression

Investigations on hSGLTs were performed in oocytes of the African clawfrog *Xenopus laevis*. Permission for keeping and surgery of frogs exists at the Julius-von-Sachs Institute and is registered at the Regierung of Unterfranken (#70/14). Mature female *Xenopus laevis* frogs were anesthetized by immersion in water containing 0.1% 3-aminobenzoic acid ethyl ester. Following partial ovariectomy, stage V or VI oocytes were treated with 0.14 mg/ml collagenase I in Ca^2+^-free ND96 buffer (10 mM HEPES pH 7.4, 96 mM NaCl, 2 mM KCl, 1 mM MgCl_2_) for 1.5 h. Subsequently, oocytes were washed with Ca^2+^-free ND96 buffer and kept at 16°C in ND96 solution (10 mM HEPES pH 7.4, 96 mM NaCl, 2 mM KCl, 1 mM MgCl_2,_ 1 mM CaCl_2_) containing 50 mg/l gentamycin. Selected oocytes were injected with 25 ng cRNA of SGLT1, SGLT3 and mutants, respectively using a Nanoject II Auto Nanoliter Injector (Drummond Scientific Company, Broomall, USA). For heterologous expression of transporters oocytes were incubated for 4 to 5 days at 16°C in ND96 solution containing gentamycin.

### Electrophysiology

For two-electrode voltage-clamp experiments, oocytes were perfused with a standard-solution containing 10 mM MES/Tris pH 5.0, 30 mM NaGluconate, 2 mM KGluconate, 1 mM MgGluconate_2_, and 2 mM CaGluconate_2_, 100mM D-sorbitol. Osmolality was adjusted to 220 mosmol/kg using D-sorbitol. Due to the small SGLT-derived currents, gluconate instead of chloride salts were used to avoid disturbances of oocyte endogenous currents. Based on the standard solution, substrate containing solutions were prepared without affecting the ionic strength and osmolality via exchanging D-sorbitol with the respective concentration of the substrate (e.g. when preparing a 100 mM D-glucose solution, 100 mM D-sorbitol was replaced by 100 mM D-glucose). Solutions with varying sodium concentration were prepared by mixing a sodium chloride-based solution (10 mM MES/Tris pH 5.0, 100 mM NaGluconate, 2 mM KGluconate, 1 mM MgGluconate_2_, and 2 mM CaGluconate_2_) with a NMDG-based solution (10 mM MES/Tris pH 5.0, 100 mM NmdgGluconate, 2 mM KGluconate, 1 mM MgGluconate_2_, and 2 mM CaGluconate_2_). Osmolality was adjusted to 220 mosmol/kg using D-sorbitol. Determination of the substrate-induced currents and voltage changes was done in *Xenopus laevis* oocytes using the two-electrode voltage clamp technique using a commercial amplifier (TURBO TEC-10CX, npi electronics, Tamm, Germany) connected to a personal computer via a low-noise 16 Bit AD/DA-USB 2.0 computer interface (LIH 8+8, HEKA Instruments Inc,. Lambrecht/Pfalz, Germany). Voltage stimulation and data acquisition was done with the multi-channel data acquisition software Patchmaster (HEKA Instruments Inc,. Lambrecht/Pfalz, Germany). The borosilicate glass capillaries for oocyte impalement (TW120F-3, World Precision Instruments Inc., Sarasota, USA) were pulled with a vertical capillary puller (Scientific Instrument Lab, Narishige Japan), filled with 3 M KCl and had a resistance of around 0.5 MΩ. The reference electrode (filled with 3M KCl and closed with a 1% Agar plug) was placed in the bath medium close to the oocyte. Voltage clamp experiments were performed as described elsewhere [24]. Holding potential and test potential was set to -50 mV during the entire experiment. For current clamp measurements the amplifier was set to the current clamp mode. The current was clamped to zero and the free-running membrane potential was monitored.

Steady state current measurements in the voltage clamp configuration in response to substrate application were performed at a membrane potential of -50 mV. Substrate-induced currents were determined by subtracting steady-state currents in the absence of substrate from steady-state currents in the presence of substrate [25]. For the determination of substrate affinities, the currents were plotted as a function of the applied substrate concentration and fitted with a Michaelis-Menten equation of the form: I = I_max_^S^ [S]/([S] + K_0.5_^S^. In H_2_O-injected control oocytes no significant glucose-induced currents were observed.

### Protein extraction and immunoblotting

*Xenopus laevis* oocytes were injected with *in vitro* transcripts of hSGLT1 or hSGLT3 (wt, ΔM500 or ΔI501) or with H_2_O as a control. For efficient protein expression, cells were incubated for 4 days at 16°C in ND 96 solution (96 mM NaCl, 2 mM KCl, 1 mM MgCl_2_, 1 mM CaCl_2_, 5 mM HEPES, pH 7.4) and subsequently homogenized by repeated pipetting in solubilisation buffer (10mM HEPES, pH 7.9, 1mM MgCl_2_, 83mM NaCl, 0,5mM PMSF, protease inhibitor cocktail cOmplete [Roche]). Plasma membranes from homogenates of 25 oocytes were isolated by sequential centrifugations at 2x 1000g (10 min) and 10000g (20 min) at 4°C. Precipitates of the final centrifugation step were solubilized in modified Laemmli loading buffer (126 mM Tris/HCl pH 6,8, 300 mM DTT, 6% SDS, 10% Glycerol 0,2% bromophenol blue). Equal amounts of precipitated membrane fractions were subjected to PAGE and analyzed by Western immunoblots probed with polyclonal rabbit antibodies against hSGLT3 (1:500, [12]) or hSGLT1 (1:500, [26]). To control protein loading blots were probed with monoclonal mouse anti-Actin antibody (1:5000, clone C4, Millipore, Temecula, CA). For detection HRP-conjugated goat anti rabbit or anti mouse immunoglobulins (1:10000; Jackson ImmunoResearch Laboratories, West Grove, PA) were applied and after washing developed with self-prepared chemiluminescence reagent (0.1 M Tris pH 8.6, 1.25 mM Luminol, 0.6 mM p-Cumaric acid, 0.01% H_2_O_2_).

### Tracing of membrane proteins

YFP fluorescence intensity of oocytes expressing hSGLT3, hSGLT1 and mutants thereof was measured with a confocal laser scanning microscopy (Leica DM6000 CS, Leica Microsystems CMS GmbH, Wetzlar, Germany) equipped with a Leica HCX IRAPO L25x/095W objective. The optical plane was set to the equator of the oocyte and the settings for YFP fluorescence acquisition and laser intensity were kept constant for all tested oocytes. YFP-fluorescence intensity of a quarter of the oocyte was quantified using the LAS AF software (Leica Microsystems CMS GmbH, Wetzlar, Germany).

### Homology modeling

Selected SGLT related sequences (hSGLT3_NP_055042, P31636|SC5A4_PIG, sp|P11170|SC5A1_RABBIT, sp|P13866|SC5A1_HUMAN, sp|P26429|SC5A1_PIG, sp|P53791|SC5A1_SHEEP, sp|P53790|SC5A1_RAT, sp|P26430|SC5A2_RABBIT, sp|Q28610|SC5AA_RABBIT, sp|Q28728|SC5AB_RABBIT, sp|P31637|SC5A3_CANLF) were aligned to vSGLT1 (PDB:2xq2) using SALIGN ([27]; https://modbase.compbio.ucsf.edu/salign/).

A homology model of hSGLT3 (uniprot: Q9NY91) was build using vSGLT1 (PDB: 2XQ2) as a template [28]. The hSGLT3 homology model was threaded onto the vSGLT1 model (PDB: 2XQ2) using Modeller [29] via the Chimera GUI interface [30]. No further optimization or manual editing of the model was performed. Visual Molecular Dynamics (VMD) software [31] was used for visualizing and displaying homology models. 3V [32] was used to identify and visualize the intracellular cavity/channel via the web interface found at http://3vee.molmovdb.org.

### Statistical analysis

All results were gained from ≥ two independent experiments (oocyte batches from at least two different frogs). At least 2–3 oocytes from each oocyte batch were measured. Results are given as mean ± standard deviation (SD). Using Prism 6 software we tested for normal distribution of data values. According to Kolmogorov-Smirnov test, the samples were distributed normally, and thus parametric tests were appropriate for statistical studies. Significance between groups was tested using one-way ANOVA tests. Statistical significance (P) is given in the respective figure legends. For statistical analysis and graph preparations the software Igor Pro7 (waveMetrics, Inc., Lake Oswego, Oregon, USA) and Excel (Microsoft Corp. Redmond, Washington, USA) was used.

## Results

### Whole-exome sequencing of ADHD patients reveals a mutation in human SGLT3

To identify genetic variants that may be related to the ADHD phenotype whole-exome sequencing (WES) was conducted in several multi-generational families of German descent with high density of patients affected with ADHD. WES data of two diseased individuals and subsequent genotyping of family members disclose a triple nucleotide deletion in *SLC5A4* on chromosome 22q12.3 (Fig 1A). As revealed by conventional and quantitative RT-PCR, transcripts of *SLC5A4* were found in various human brain regions (S1 Fig). This gene encoding the sodium-glucose transporter SGLT3 consists of 15 coding exons and within the 13^th^ exon nucleotides 1498–1500 were deleted (c.1498-1500delATG), which results in the extinction of a methionine at position 500 in transmembrane segment 11 (TM11) of the protein (p.[ΔM500]; Fig 1B). When we analyzed the entire family for this mutation by Sanger sequencing, only imperfect segregation of the methionine deletion with the categorical ADHD diagnosis was detected (Fig 1A). Whereas five patients display the mutation in the *SLC5A4* gene another three individuals (Fig 1A) do not show a match between geno- and phenotype.

Just as much, the structural and functional aspects of this mutation are important in order to evaluate the negative effect this mutation might have on a human carrier. In SGLT3 methionine at position 500 [ΔM500] is located in TM11 which was shown to be critical for proper function of SGLT1 and SGLT2. Mutations in TM11 of the two structurally related sodium-glucose transporters disrupt sugar translocation and cause intestinal and renal diseases, respectively [8, 33]. Considering the unique property of SGLT3 to only sense and not transport glucose the question arises whether changes in TM11 have the same dramatic effects as in the homologous SGTL isoforms 1 and 2.

### Functional properties of hSGLT3 expressed in *Xenopus laevis* oocytes

**Fig 2:**
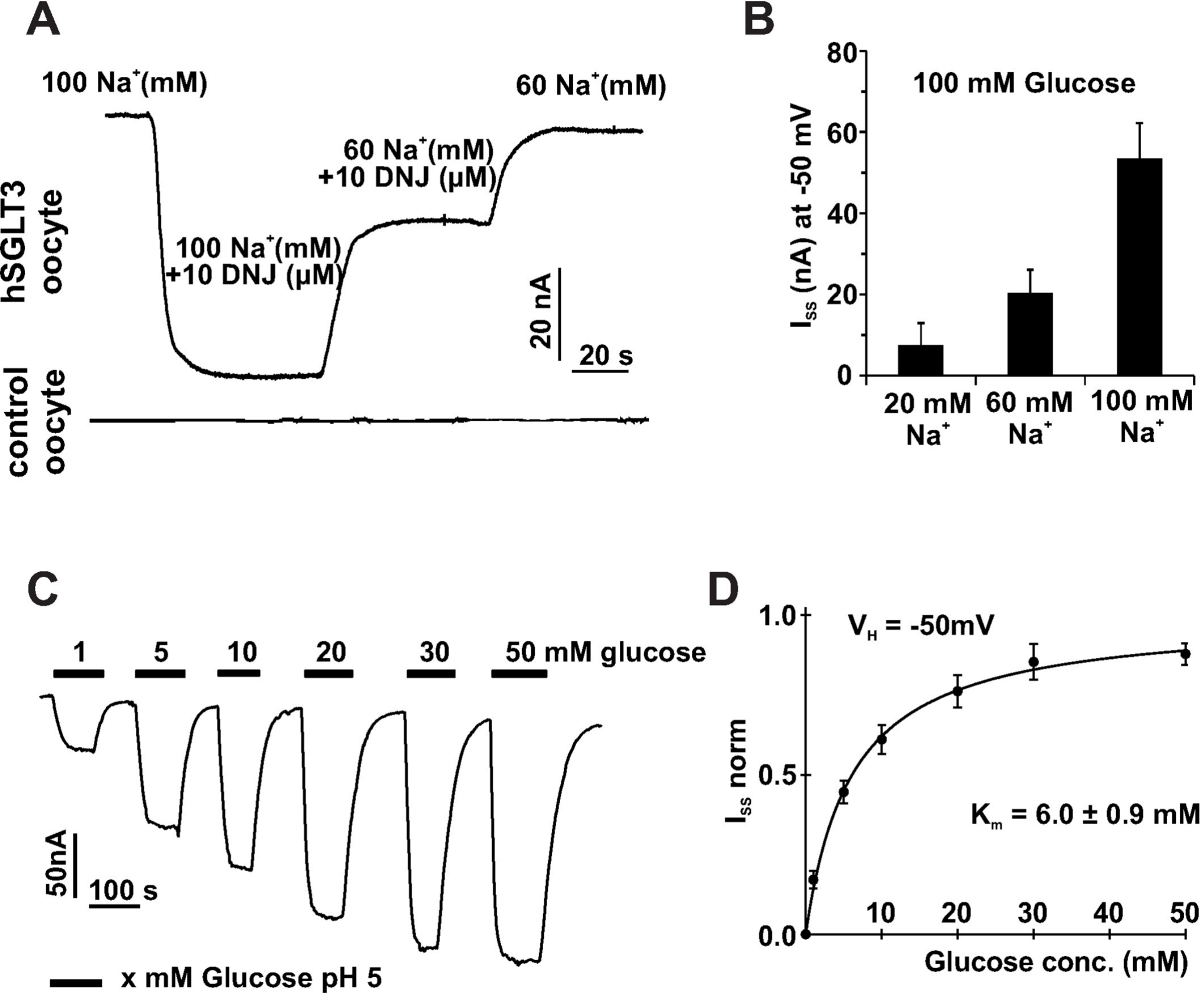
Dependence of SGLT3 currents on external sodium and glucose concentrations. (A) Two-electrode-voltage clamp recordings (V_H_ = -50 mV) from *Xenopus laevis* oocytes injected with hSGLT3 display DNJ-induced inward currents that decrease by reduction of the external sodium concentration. Representative current traces from oocytes injected with hSGLT3 or water (control oocytes) are shown. (B) Bar graph quantifies hSGLT3 currents induced by 100mM glucose at different external sodium concentrations (n = 6 ± SD of two independent experiments). (C) Continuous recordings from oocytes expressing hSGLT3 display sodium inward currents that increase in amplitude by elevating the external concentration of D-glucose as indicated (V_H_ = -50 mV; [Na^+^]_e_ = 30 mM; pH 5). (D) Dose-response curve of hSGLT3 currents activated by different glucose concentrations as recorded in panel C. Data were fitted with Michaelis-Menten equation resulting in a K_0.5_-value of 6.0±0.9 mM (n = 6 ± SD of two independent experiments).

Human SGLT3 is unable to transport carbohydrates into the cytoplasm but instead evokes sodium inward currents upon binding of sugar molecules [12]. To explore fundamental properties of the glucose sensor, we perform two-electrode voltage clamp recordings from *Xenopus laevis* oocytes injected with cRNA of hSGLT3[wt]. Substantial Na^+^ currents were recorded (V_H_ = -50 mV) upon perfusion of oocytes with 10 μM DNJ (1-desoxynojirimycin), an imino sugar that specifically activates hSGLT3 ([34], Fig 2A) but did not evoke a current response in control oocytes. It is essential to record under mild acidic conditions (pH 5.0) as proton concentrations in the neutral range almost fail to evoke sodium currents by hSGLT3 [12]. Upon reduction of external Na^+^ concentration from 100 mM to 60 mM current amplitude decreased by [65%] and returned to baseline after washout of the agonist (Fig 2A). Under the same conditions current amplitudes in response to 100 mM glucose were quantified at different external sodium concentrations. In the presence of glucose, currents of hSGLT3 increased with elevated external sodium concentrations (n = 6 ± SD of two independent experiments; Fig 2B), which is in line with the sodium dependence of currents induced by DNJ.

To explore the activation constant for glucose, oocytes expressing hSGLT3[wt] were recorded under voltage clamp conditions at a defined membrane potential of V_H_ = -50 mV. Perfusion of oocytes with recording buffer (30 mM Na^+^) supplemented with glucose between 1 mM and 50 mM resulted in augmentation of current amplitudes with increasing glucose concentrations (Fig 2C). When plotting the obtained current amplitudes as a function of the applied glucose concentration, we could describe the resulting saturation curve with a Michaelis-Menten equation. Calculation of data revealed a K_0.5_ value of 6.0 ± 0.9 mM for the activation of hSGLT3 by glucose (Fig 2D).

### Inactivation of hSGLT3 and hSGLT1 by WES-derived mutation

As methionine 500 in hSGLT3 was found to be deleted in a family densely affected with ADHD, the question arose if the mutated transporter is still functional. Moreover, experiments with this mutation in TM11 outside the core structure (TM1–TM10) of sodium transporters [7] are adequate to verify the importance of such adjacent domains for fundamental function of transporters.

**Fig 3:**
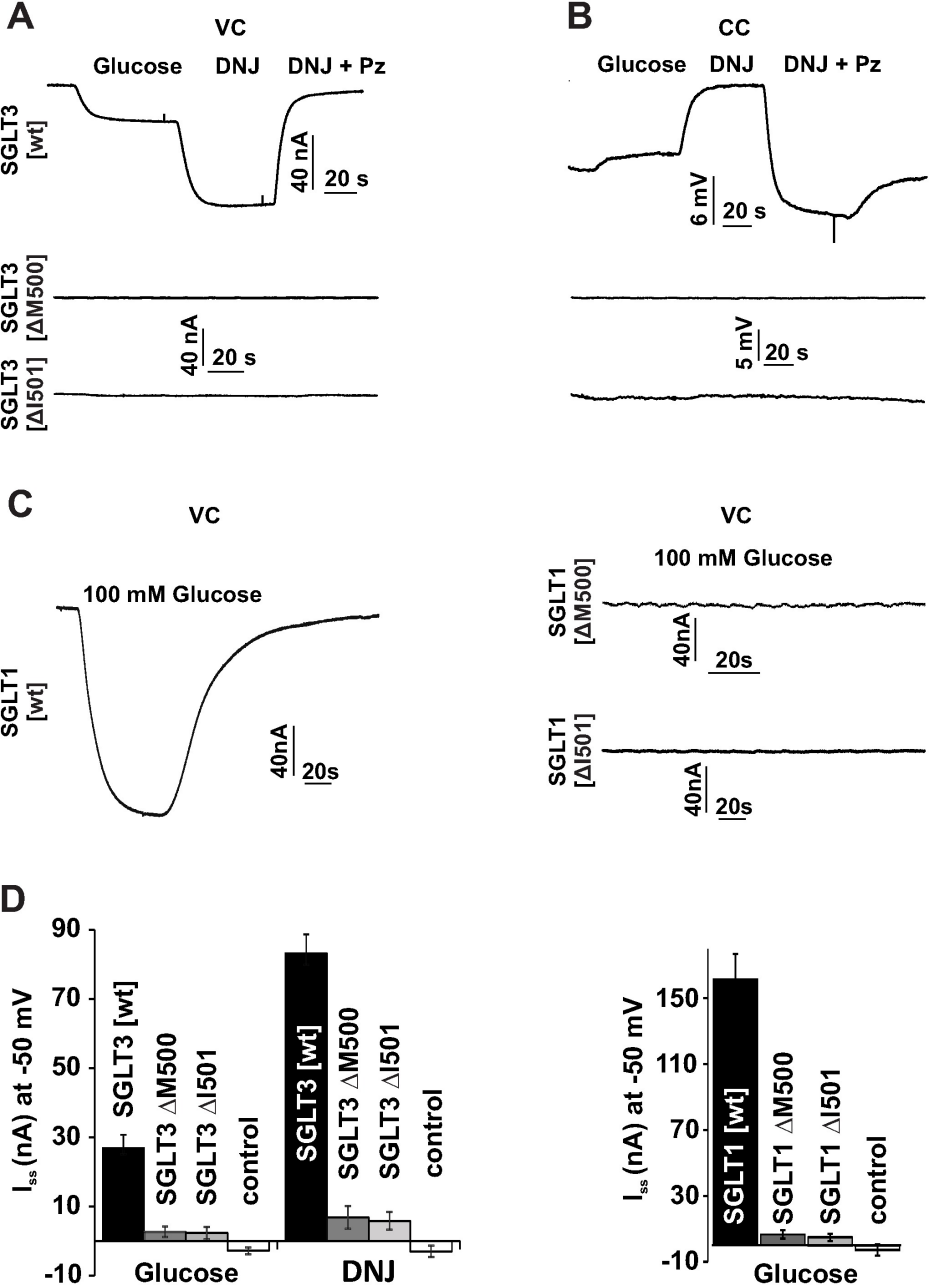
Loss of function by deletion of M500/I501 in hSGLT3 and hSGLT1. (A and B) *Xenopus laevis* oocytes injected with cRNA of hSGLT3[wt], hSGLT3[ΔM500] and hSGLT3[ΔI501] were recorded in the voltage clamp mode (VC, panel A) and in the current clamp mode (CC, panel B). Representative continuous recordings at a holding potential of –50 mV, 30 mM Na^+^ and pH 5 from oocytes expressing hSGLT3[wt] display sodium currents in response to D-glucose (75 mM) and Deoxynojirimycin (DNJ; 10μM). Currents were blocked by the specific inhibitor Phlorizin (Pz; 240 μM). Current clamp recordings of the same oocyte reveal D-glucose- and DNJ-induced depolarization as well as phlorizin-induced hyperpolarization of the cell. Using identical conditions, no current or voltage responses were observed in oocytes expressing hSGLT3[ΔM500] or hSGLT3[ΔI501]. (C) Oocytes injected with cRNA of hSGLT1[wt], hSGLT1[ΔM500] and hSGLT1[ΔI501] were measured in the absence and presence of 100 mM glucose (pH 5, 30 mM Na^+^ and a membrane potential of -50 mV). Oocytes expressing hSGLT1[wt] display sodium currents in response to glucose, whereas no responses were observed in oocytes expressing hSGLT1[ΔM500] or hSGLT1[ΔI501]. (D) Left panel: bar graph quantifies current amplitudes in response to either D-glucose (75 mM) or DNJ (10 μM) at -50 mV and 30 mM Na^+^. As a negative control, results from H_2_O-injected oocytes are displayed (n = 6 ± SD of two independent experiments). Right panel: bar graph quantifies current amplitudes in response to D-glucose (100 mM) at -50 mV and 30 mM Na^+^ of hSGLT1[wt], hSGLT1[ΔM500] and hSGLT1[ΔI501] and water controls (n = 6 ± SD of two independent experiments).

Using the *Xenopus laevis* oocyte expression system, recordings in the current clamp mode and in the voltage clamp mode were performed with hSGLT3[wt] as a control (Fig 3A, 3B and 3D). Continuous recordings under voltage clamp conditions (V_H_ = -50mV) revealed robust activation of Na^+^ currents by successive application of glucose (75 mM) and DNJ (10μM) with a 3-fold higher efficacy of the synthetic agonist (Fig 3A and 3D; n = 6 ± SD of two independent experiments; p = 0.001). Co-application of the SGLT-specific antagonist phlorizin [35] completely reversed the activation of sodium currents (Fig 3A). Similarly, when these drugs in the same order were applied under current clamp conditions, we observed a stepwise increase of the membrane potential with glucose and DNJ, which returned to more negative potentials by the effect of phlorizin (Fig 3B).

Although TM11 of SGLT3 is not located within the functional core structure, a deletion of an amino acid at the boundary of the lipid bilayer to the extracellular side may have dramatic functional effects. We generated the hSGLT3[ΔM500] mutant by site-directed mutagenesis and analyzed its electrophysiological properties with the same protocol we executed on hSGLT3[wt] (Fig 3A, 3B and 3D). In contrast to the wildtype, negligible responses were observed with hSGLT3[ΔM500] when recorded in *Xenopus laevis* oocytes under current clamp and voltage clamp conditions with both agonists (Figs 3A, middle panel and 3B and D).

Our WES study revealed patients of ADHD with heterozygous genotypes. To better understand the observed phenotype and to elucidate whether the phenotype is due to haploinsufficiency or the mutation has a dominant negative effect over the expression of SGLT3, we either expressed hSGLT3[wt] alone or co-expressed hSGLT3[wt] and hSGLT3[ΔM500]. Oocytes injected with cRNA of both, hSGLT3[wt] and hSGLT3[ΔM500] evoked the same glucose-induced current amplitude as oocytes injected with equal amount of wt cRNA only (S2 Fig). Thus, the mutant carrier/sensor has no dominant negative effect. In other words, under heterozygous conditions, hSGLT3 derived from the healthy allele is not affected by hSGLT3 synthesized from the mutated allele.

To explore if this loss of function is a matter of structural disturbance or a missing function by the amino acid itself, we engineered another mutant with a deletion of the adjacent residue at position 501, hSGLT3[ΔI501]. But again, when recorded in *Xenopus laevis* oocytes this second mutant failed to evoke substantial currents or voltage changes upon application of glucose or DNJ (Fig 3A and 3B, lower panel, Fig 3D for quantification).

Previously, in the glucose transporter hSGLT1 a substitution of arginine 499 by histidine in TM11 was found to reduce glucose-induced currents and transport of carbohydrates by more than 50% [8]. Apparently in hSGLT1 the amino acid exchange is partially tolerated and thus we wondered if deletions that heavily eliminate hSGLT3 function, have less severe effects on the hSGLT1 isoform.

To monitor sodium currents of hSGLT1, oocytes were injected with transcripts of hSGLT1[wt], hSGLT1[ΔM500] and hSGLT1[ΔI501], respectively. Upon two-electrode voltage clamp recordings (V_H_ = -50 mV) glucose–induced sodium currents with an amplitude of 161 ± 15.27 nA (n = 6 ± SD of two independent experiments, Fig 3D) were observed from wt-injected cells. However, no currents were recorded from cells expressing the mutants hSGLT1[ΔM500] and hSGLT1[ΔI501] (Fig 3C and 3D).

Thus, regardless of different functional properties, both the glucose transporter hSGLT1 and the glucose sensor hSGLT3 entirely lose their activity upon deletion of amino acids being located in TM11 close to the extracellular space.

### Membrane targeting of hSGLT3[ΔM500] is impaired

The absence of any electrophysiological recordings in cell systems may have two main reasons. On the one hand the protein is not synthesized or alternatively it is not translocated to the membrane (and therefore does not conduct charged particles). In both cases no currents would be observed. To investigate if protein processing is responsible for the absence of currents in oocytes injected with transcripts of hSGLT mutants we conducted Western immunoblots and fluorescence imaging with *Xenopus laevis* oocytes.

**Fig 4:**
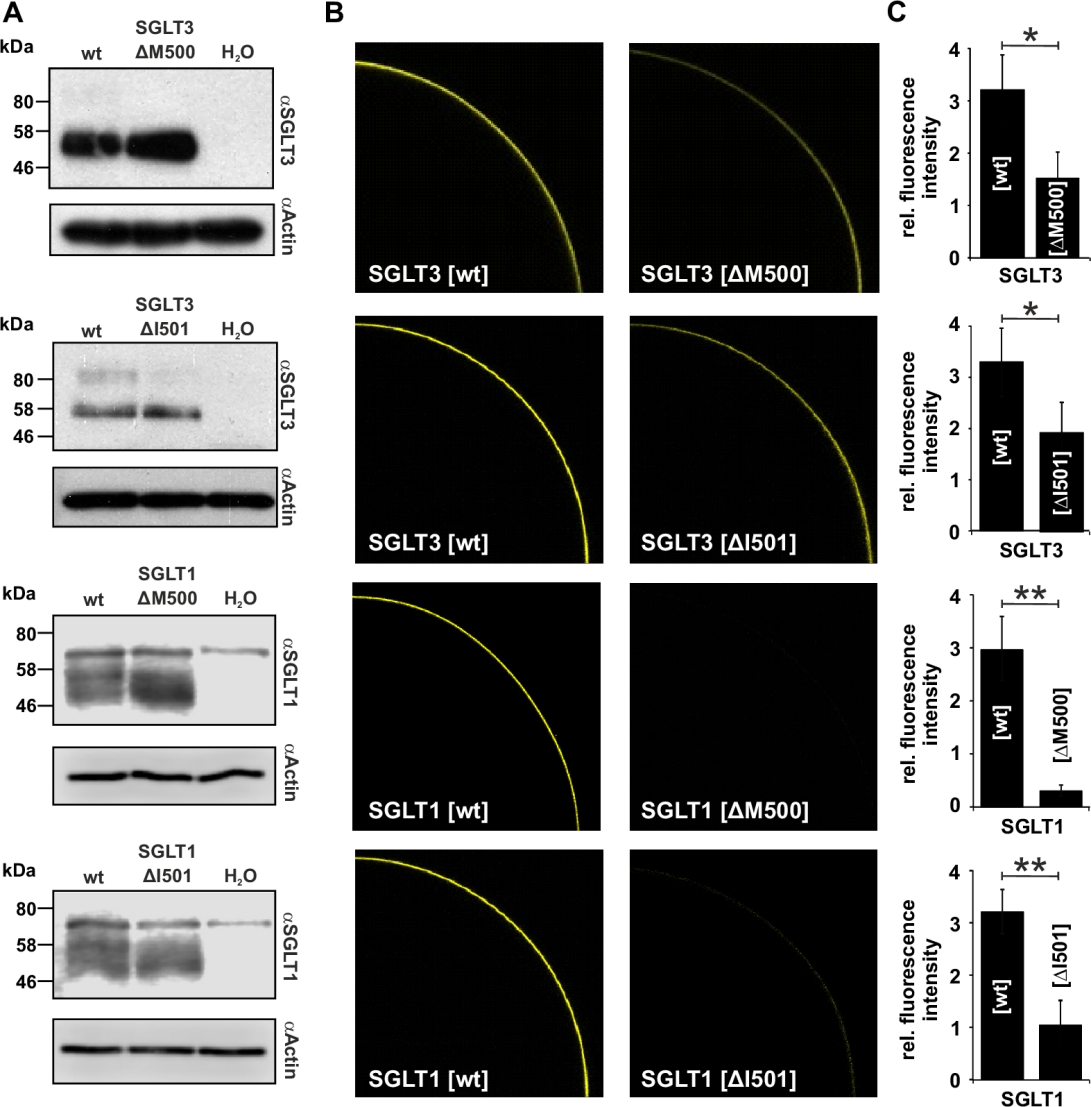
Targeting of hSGLT3 and hSGLT1 to the plasma membrane is impaired by deletion of M500 or I501. (A) Crude membrane fractions of *Xenopus laevis* oocytes injected with cRNA of wildtype and mutated hSGLTs or H_2_O (control) were analyzed by western immunoblotting. As revealed by specific hSGLT3 and hSGLT1 antibodies, signals of wildtype and mutant injected oocytes were almost identical with no specific signal in water-injected oocytes. A single protein band present in samples and control (αSGLT1) are due to cross-reactivity of the antibody (compare S3 Fig). Loading of identical amounts of protein was controlled by detection of endogenous actin with the appropriate antibody (one representative experiment out of 2 independent experiments is shown). (B) Representative confocal laser scanning images of *Xenopus laevis* oocytes injected with YFP-tagged hSGLT constructs are shown. Membrane fluorescences of the mutated hSGLT1 and hSGLT3 are substantially lower as compared to wildtype. (C) Bar graphs show the fluorescence intensity of the mutants hSGLT[ΔM500] or hSGLT[ΔI501] as proportion of the respective wt fluorescence (n = 6 ± SD of two independent experiments, * indicate p < 0.01; ** indicate p < 0.001).

When subjected to Western immunoblot, crude membrane fractions of oocytes injected with identical amounts of cRNA of wildtype and mutated transporters display specific signals that are absent in H_2_O-injected controls. For hSGLT3 and hSGLT1 the expected molecular weights are 72.4 kDa and 73.4 kDa, respectively. However, the majority of specific signals were found in the range of 58 kDa, which most probably results from proteolytic degradation during preparation of membranes. Simultaneous analysis and comparison of hSGLT1/3[wt] with their corresponding mutants document nearly identical amounts of protein in both preparations. (Figs 4A and S3).

Thus, production of SGLT proteins is not affected by the deletion of amino acids and in addition mutated transporters were still located in entire membrane fractions as almost no signals were found in cytosolic fractions of preparations (data not shown). However, the question remains if the mutated transporters were properly targeted to the plasma membrane. To monitor this capability, we generated SGLT:YFP fusion constructs and heterologously expressed them in *Xenopus laevis* oocytes. Prior to fluorescence imaging we tested the functional properties of fusion constructs by two electrode-voltage recordings and did not find any difference to “native” SGLTs (S2 Fig). Upon confocal laser scanning microscopy both, hSGLT1[wt] and hSGLT3[wt] fusion constructs were detected at the equator of oocytes with similar fluorescence intensity (Fig 4B). In comparison to the wildtype, membrane fluorescences of the mutants hSGLT3[ΔM500] and hSGLT3[ΔI501] were reduced by 53% (n = 6 ± SD of two independent experiments; p <0.01) and 42% (n = 6 ± SD of two independent experiments; p < 0.01), respectively (Fig 4C). Interestingly, the identical mutations in SGLT1 affected the fluorescence intensity even stronger with reductions between 68% and 91% (n = 6 ± SD for each mutant/wt of two independent experiments; p < 0.001) as compared to the wildtype (Fig 4B and 4C). These results indicate that deletions of single amino acid residues in TM11 of both hSGLTs markedly influence the trafficking of the proteins to the plasma membrane. Although the fluorescence intensity at the oocyte’s periphery of the hSGLT3 mutants was reduced by around 50% only, sodium currents or membrane depolarization were reduced by more than 90% (Fig 3A, 3B and 3D).

### SGLT3[ΔM500] from a structural perspective

**Fig 5:**
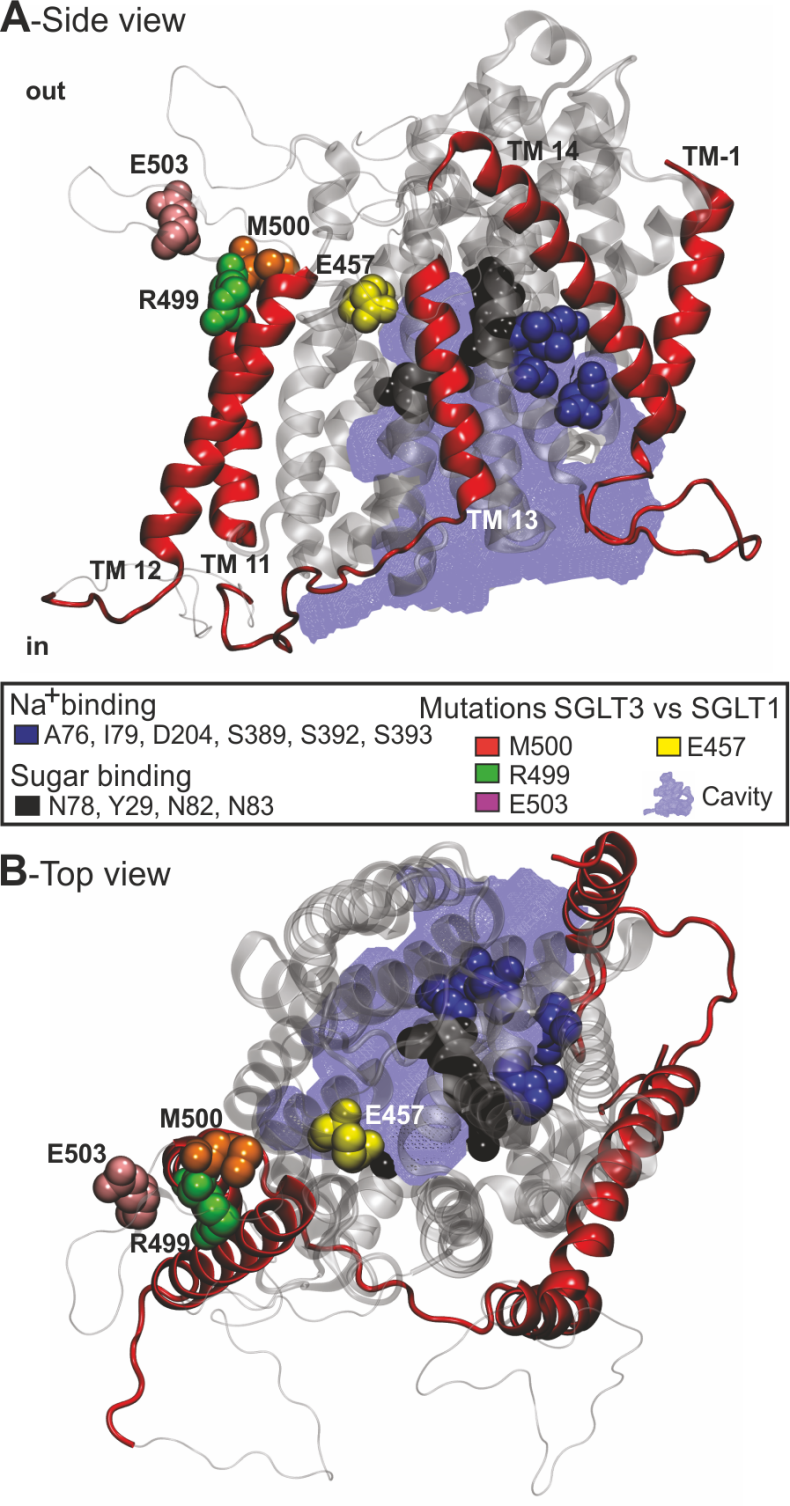
A structural perspective of the ΔM500 deletion found in hSGLT3. (A and B) Homology model of hSGLT3 that show the localization of mutated residues M500 (orange sphere), E457 (yellow sphere), R503 (pink sphere) and R499 (green sphere) and residues involved in Na^+^ (blue spheres) and sugar binding (black spheres) from a side (A) and top (B) perspective. Core TM helices 1–10 are shown in transparent silver whereas the surrounding helices are red and loop regions not supported by the model template are shown as silver strings. The modelled hSGLT3 carrier cavity/vestibule is shown in blue as a mesh structure.

To visualize the position of the deletion ΔM500 in hSGLT3 and provide a structural perspective of the ADHD-derived mutation, we generated a 3D homology model of hSGLT3 based on the vSGLT crystal (PDB: 2XQ2; Fig 5; [28]). The sequence alignment of vSGLT and hSGLT3 (S4 Fig), which was used to generate a homology model of hSGLT3, revealed an overall identity of 23%.

The alignment (S4 Fig) and the homology model (Fig 5) revealed that M500 is situated on TM11 (TM-1 and TM11-TM14 are marked in red, Fig 5) that is not part of the SGLT core structure (TM1 to 10, marked in transparent silver, Fig 5A). TM11 is positioned parallel to TM10 and TM12 thereby positioning M500 opposite the previously reported sodium and sugar binding residues (marked in blue and black, respectively, Fig 5; [28]). How the deletion of either M500 or I501 influences the position of TM11 and thereby the overall structural organization of hSGLT3 remains to be shown.

## Discussion

### Disruptive rare variant of SLC5A4 may be involved in genetic risk for ADHD

ADHD has a high genetic risk component, and according to large twin and family studies heritability has consistently calculated to be approximately 80% [36, 37]. Genome-wide screening approaches and candidate gene studies have attempted to identify ADHD-associated risk genes, but so far no single variant has passed the significance threshold of replicable gene-disorder association [38, 39, 40]. The SNP heritability of ADHD has been estimated at 0.28 indicating that common SNPs contribute substantially to ADHD susceptibility [41], although each locus exerts only a very small effect (OR<1.1). The contrast to the overall heritability of ADHD suggest that the genetic component of ADHD cannot be explained only by common genetic variants. Thus, presumably also rare variants contribute to the ADHD risk and account for the missing part of heritability. Previously, one exome sequencing study identified rare variant that might predispose to ADHD, although causality was not proven [42].

Upon network analysis ADHD-associated genes were found to be enriched within specific functional categories. Based on protein-protein interaction a network was constructed showing that genes involved in biological processes such as synaptic transmission, catecholamine metabolism, G-protein signaling pathways and cell migration were over-represented in ADHD [43, 44]. Although a putative category like energy homeostasis is not emphasized by this network analysis, there are conclusive concepts that explain characteristic symptoms of ADHD such as inattention or deficits in working memory function by energetic insufficiency in neurons [45]. Recently, common and rare variants of the glucose-transporter-3 gene (SLC2A3) were found to associate with ADHD and upon testing patients displayed deficits in cognitive processing [18]. In line with this notion hSGLT3 as a glucose sensor [12] localized in various regions of the human brain (S1 Fig) is an appropriate candidate for the concept of deficient energy balance in ADHD neuropathology.

In our WES approach in ADHD families we identified a triplet deletion in *SLC5A4*, suggesting profound functional deficits of hSGLT3. However, the co-segregation of the *SLC5A4* variant was imperfect. Two family members, one female individual (ID6) and her son (ID15) carry c.1498-1500delATG but are not affected by ADHD. This may indicate incomplete penetrance of the variant, which is commonly observed in neurodevelopmental disorders, such as 22q11.2 deletion syndrome [46]. One ADHD patient (ID19) in this family, who has been diagnosed with childhood ADHD, is not a carrier of c.1498-1500delATG. Behavioural dysfunction with similarities to ADHD but a different neurodevelopmental pathway resulting in a phenocopy are encountered frequently in core families with several sibling and one or both parent(s) affected with ADHD. Here, a severely affected mother with two more children suffering from ADHD likely associated with early pathogenic care, characterized e.g. by early separation, frequent change in caregivers, institutionalisation or neglect, may lead to severe global psychosocial and cognitive dysfunction and deviant developmental trajectories of brain maturation in terms of a reactive attachment disorder (RAD) [47]. Despite the apparent dominant segregation pattern, ADHD is not a monogenic disorder in the pedigree investigated. WES revealed other rare SNVs in this family, suggesting that several genes may carry risk variants for ADHD in each of those. Based on the WES data, we did not expect a single gene to be associated with the clinical phenotype nor perfect co-segregation pattern of the rare, non-reference allele with ADHD in subsequent segregation analyses. A main contributing factor to the observed patterns may be incomplete penetrance and phenocopies, which is common in ADHD. Although we were quite liberal in selecting a rare variant for further analysis (through including suggestive linkage signals), the observed pattern is similar to findings in WES studies of ADHD [20] and other neurodevelopmental disorders (e.g. autism spectrum disorders, [48]).

### hSGLT3 TM11 is critical for trafficking and thus functionality

Here we characterized the basic features of SGLT3 in the heterologous expression system of Xenopus *laevis* oocytes. We confirmed that SGLT3 mediates sodium specific currents in the presence of glucose and DNJ that were abolished following phlorizin treatment. These results are well in line with findings of Diez-Sampedro et al. [12] and Voss et al. [34].

X-ray analysis of crystals from vSGLT revealed a core structure of 10 transmembrane helices (TM1-TM10) that could be associated with substrate and sodium binding and thus TMs 1 to 10 are particularly important for the transport activity of SGLTs (Fig 5; [6, 7, 49, 50]). This core structure is flanked by a single transmembrane segment on the amino terminal side and three TMs at the carboxy terminus, named -TM1 and TM11-13, respectively (according to [7, 51]). Several amino acids were shown to be important for proper function of SGLTs as mutation caused intestinal and renal disease. Martin et al. [8] identified 31 mutations of SGLT1 in 25 families suffering from glucose-galactose malabsorption (GGM). Among the mutations in SGLT1, the mutation R499H was found in TM11. Heterologous expression combined with sophisticated electrophysiological analysis revealed that the mutation R499H decreased the current densities and lowered the substrate affinity from 0.3 mM in the WT to 2.4 mM in the mutant of hSGLT1. Moreover, via measuring charge movements and thereby determining Q_max_ of hSGLT1[wt] in comparison to the mutant R499H, the authors could demonstrate that the mutation impairs the trafficking of the transporter to the plasma membrane. This finding is well in line with our results gained by fluorescence intensity measurements of YFP-fused hSGLTs and mutants thereof (Fig 4B and 4C). Both deletions, M500 and I501, in hSGLT1 and 3 markedly decreased the fluorescence within the plasma membrane while the amount of expression probed with Western blots remained similar between WTs and mutants (Fig 4A). This indicates that the deletion mutants of hSGLT3 and particularly of hSGLT1 are impaired in plasma membrane trafficking in *Xenopus laevis* oocytes. In contrast to the mutant hSGLT1[R499H] [8], that was still able to transport glucose albeit with lower current amplitudes, the deletion mutants M500 and I501 in hSGLT1 and 3 did not respond to the application of substrates at all (Fig 3). This may indicate that in contrast to the exchange of R499 by a histidine residue, the deletion of adjacent residues M500 and I501 in TM11 (see Fig 5 for illustration) severely influence the trafficking and maybe also the function of SGLT proteins. However, given the resolution of light microcopy, the thickness of the plasma membrane in combination with the non-transparent oocytes’ cytosol, insertion of the YFP-tagged SGLT3 (and SGLT1) proteins into the plasma membrane cannot be proven by this technique. Thus, conclusions about the functionality of the SGLT1 and 3 deletion mutants remain speculative.

Although the insertion of hSGLT3 deletion mutants into the oocytes membrane could not be directly proven by light microscopy (Fig 4), previous studies of hSGLT1 indicate that TM11 severely impact on the function of the transporter [8, 52]. According to our model, M500 is in proximity to residue E457 (yellow sphere on TM10, Fig 5) that is conserved as a glutamate in SGLT3s and as a glutamine in SGLT1s. Mutation of glutamine 457 in hSGLT1 to a glutamate (as found in SGLT3s) changes the transporter from a sodium/sugar transporter to a sugar-sensor like hSGLT3 [12, 53, 54]. Therefore, a deletion at position M500 or I501 might also influence the critical residue E/Q457 and thus point to an impaired functionality of the deletion mutants in addition to their impaired membrane targeting.

Adjacent to M500 are the two hSGLT1/3 conserved residues hSGLT3-R499 and hSGLT3-E503 that were mutated and analyzed for their involvement in substrate binding, transport activity and the hSGLT1 transport mechanism [52]. hSGLT3-R499 is located on TM11 and facing TM12 towards hSGLT3-H525 (not highlighted in the model). Interestingly, mutation of hSGLT1-R499 to hSGLT1-C499 did not change hSGLT1s apparent affinity for glucose but drastically increased it for 6-deoxy-D-glucopyranose [52]. Furthermore, the transport activity of hSGLT1-C499 was only 19±1% of WT. Based on the position in the homology model and chemical nature of R499, it is likely to be an indirect effect on the structural integrity rather than a direct sugar binding effect. In the hSGLT3 homology model, E503 (violet, Fig 5) is positioned in the loop region between TM11 and TM12, in parts of the homology model that is not covered by the vSGLT1 template (2xq2). Mutation of hSGLT1-E503 to hSGLT1-C503 do not have an effect on hSGLT1 substrate specificity or transport mechanism but decreases the uptake activity to 12 ± 3% relative to wt [52]. More drastically, the hSGLT3-M500 and hSGLT3-I501 deletion mutants described in the current study lose all sugar induced Na^+^ conductance. It is likely that the deletion of M500 and M501 compromise the helical structure of TM11 and/or its interaction with TM10 and TM12 that are in close proximity. How the deletion of either M500 or I501 influences the overall structural integrity of hSGLT3 and how this may impair the translocation of the protein to the plasma membrane awaits further study. However, the previous study of hSGLT1 and the current study of hSGLT3 highlight that the non-core TM helices and especially the residues M500 and I501 are crucial for proper targeting/functioning of hSGLT3. Whether and how transport processes via hSGLT3 are impaired in the patients, carrying the ΔM500 mutation, remains to be clarified.

## Supporting information

S1 Fig PCR analysis of human brain tissues. Bar graph shows normalized expression of SGLT3 in diverse human brain tissues as indicated. Data are presented in arbitrary units as mean ± S.E. from three individual runs of quantitative real-time PCR. General presence of hSGLT3 in the same human samples was monitored by endpoint PCR as documented by the expected 344 bp fragment in the agarose gel of the inset. Cb Cerebellum, Cx Cortex, Hi Hippocampus, Hy Hypothalamus, Je Jejunum, Mo Medulla oblongata, Nc Nucleus caudates, Pn Pons, Pu Putamen.
(PDF)

S2 Fig hSGLT3/hSGLT1 deletion mutants have no dominant negative effect on the respective SGLT wildtype transport function. Oocytes (co-)expressing the indicated constructs were measured at -50 mV in the presence of 100 mM glucose (pH 5.0). The mean of glucose-induced steady state currents from n ≥ 15 oocytes from 3 independent oocyte batches are shown.
(PDF)

S3 Fig Western immunoblots of membranes from oocyte injected with transcripts of hSGLT3 and hSGLT1. Identical uncropped blots of Fig 4A are presented. Crude membrane fractions of *Xenopus laevis* oocytes injected with cRNA of wildtype and mutated hSGLTs or H_2_O (control) were analyzed by western immunoblotting. As revealed by specific hSGLT3 and hSGLT1 antibodies signals of wildtype and mutant injected oocytes were almost identical with no signal in water-injected oocytes. Loading of identical amounts of protein was controlled by detection of endogenous actin with the appropriate antibody. Position of marker bands from blots were spotted on to x-ray films with molecular weight given on the bottom (from upper to lower). Asterisks indicate unspecific staining of SGLT1 and SGLT3 antibodies in samples and controls.
(PDF)

S4 Fig Alignment of protein sequences. This sequence alignment of vSGLT and hSGLT3 was used to generate a homology model of hSGLT3 (see Fig 5).
(PDF)
