# Supplementary figures and images for: Functional analysis of a triplet deletion in the gene encoding the sodium glucose transporter 3, a potential risk factor for ADHD
Source: PLoS One. 2018 Oct 4;13(10):e0205109. doi: 10.1371/journal.pone.0205109 (PMC6171906; doi:10.1371/journal.pone.0205109)

# hSGLT3

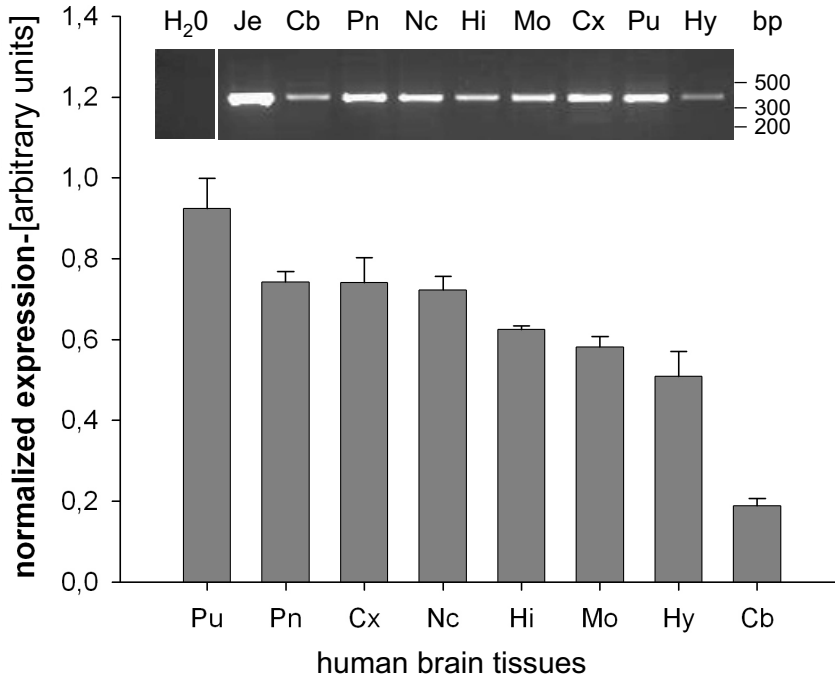

Supplement: S1 Fig — Bar graph shows normalized expression of SGLT3 in diverse human brain tissues as indicated. Data are presented in arbitrary units as mean ± S.E. from three individual runs of quantitative real-time PCR. General presence of hSGLT3 in the same human samples was monitored by endpoint PCR as documented by the expected 344 bp fragment in the agarose gel of the inset. Cb Cerebellum, Cx Cortex, Hi Hippocampus, Hy Hypothalamus, Je Jejunum, Mo Medulla oblongata, Nc Nucleus caudates, Pn Pons, Pu Putamen. (PDF) [file pone.0205109.s001.pdf]

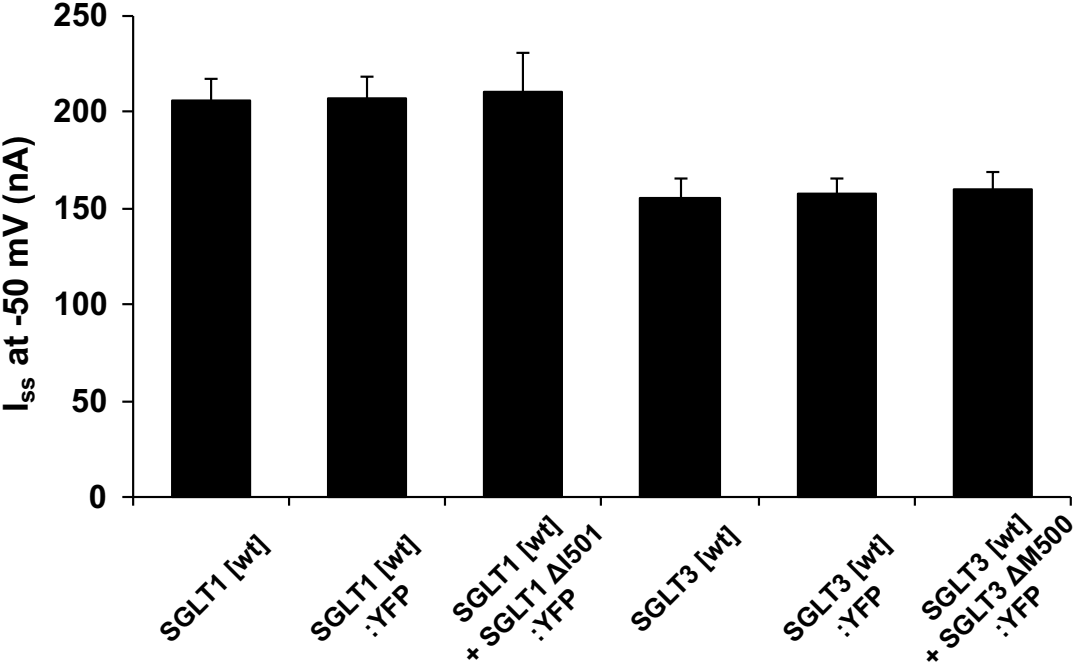

Supplement: S2 Fig — Oocytes (co-)expressing the indicated constructs were measured at -50 mV in the presence of 100 mM glucose (pH 5.0). The mean of glucose-induced steady state currents from n ≥ 15 oocytes from 3 independent oocyte batches are shown. (PDF) [file pone.0205109.s002.pdf]

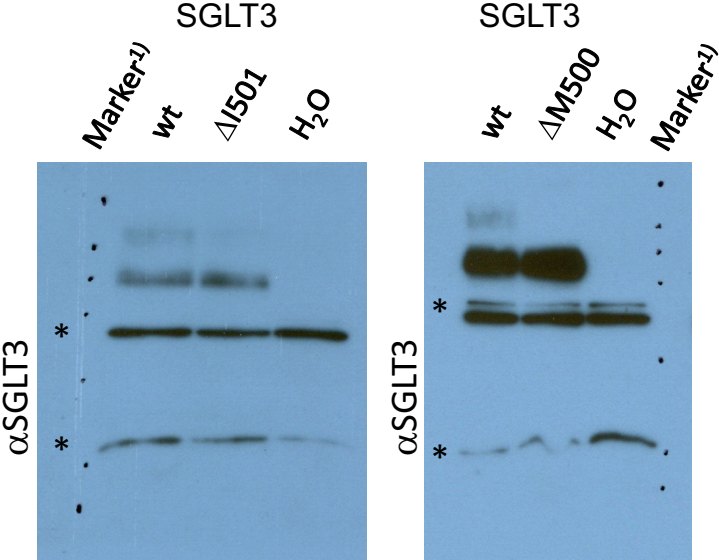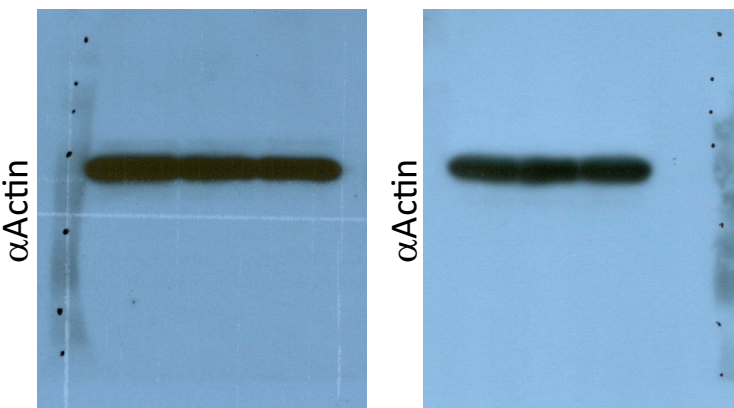

<sup>1)</sup> Molecular weight standard (kD):  
175, 80, 58, 46, 30, 23, 17

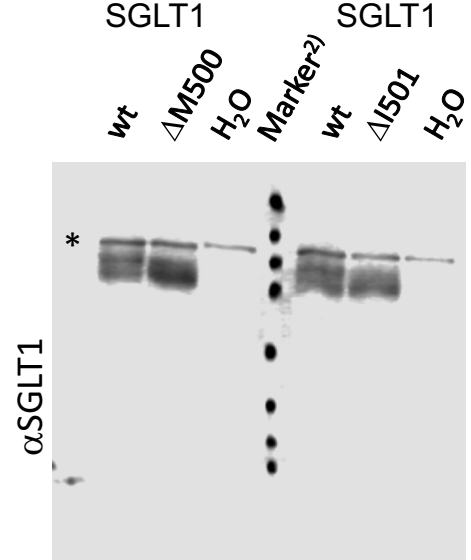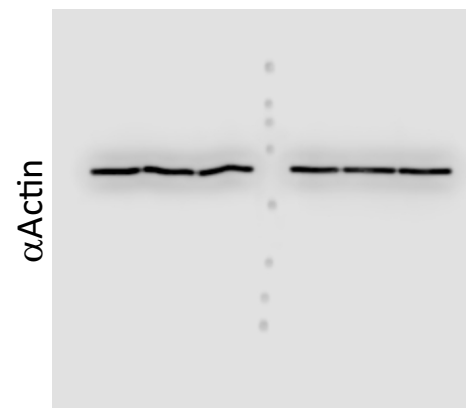

<sup>2)</sup> Molecular weight standard (kD):  
175, 80, 58, 46, 30, 23, 17, 7

Supplement: S3 Fig — Identical uncropped blots of Fig 4A are presented. Crude membrane fractions of Xenopus laevis oocytes injected with cRNA of wildtype and mutated hSGLTs or H2O (control) were analyzed by western immunoblotting. As revealed by specific hSGLT3 and hSGLT1 antibodies signals of wildtype and mutant injected oocytes were almost identical with no signal in water-injected oocytes. Loading of identical amounts of protein was controlled by detection of endogenous actin with the appropriate antibody. Position of marker bands from blots were spotted on to x-ray films with molecular weight given on the bottom (from upper to lower). Asterisks indicate unspecific staining of SGLT1 and SGLT3 antibodies in samples and controls. (PDF) [file pone.0205109.s003.pdf]
