# Supplementary material for: Functional analysis of a triplet deletion in the gene encoding the sodium glucose transporter 3, a potential risk factor for ADHD
Source: PLoS One. 2018 Oct 4;13(10):e0205109. doi: 10.1371/journal.pone.0205109 (PMC6171906; doi:10.1371/journal.pone.0205109)
Supplement: S4 Fig — This sequence alignment of vSGLT and hSGLT3 was used to generate a homology model of hSGLT3 (see Fig 5). (PDF) [file pone.0205109.s004.pdf]

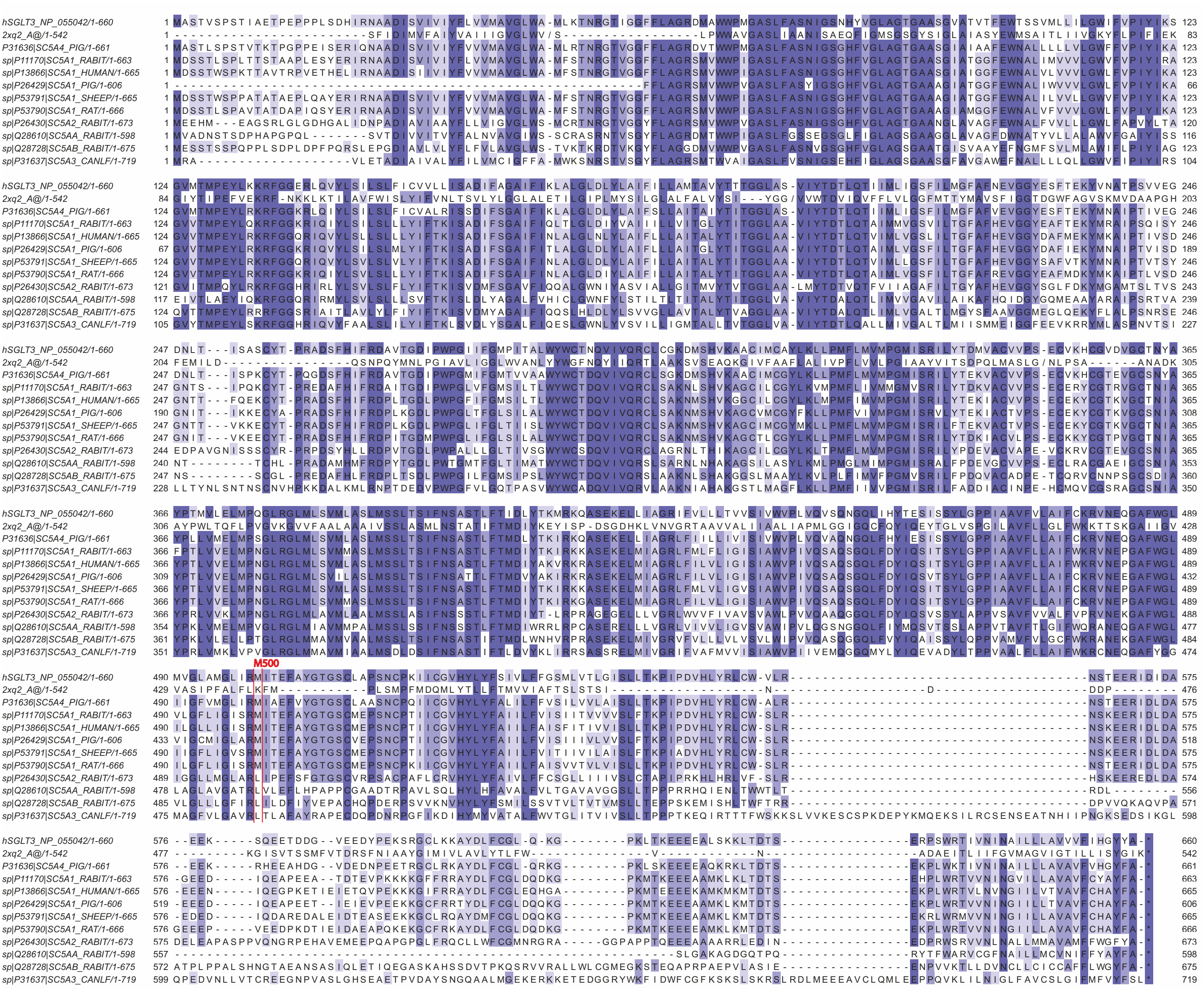
hSGLT3_NP_055042/1-660
2xq2_A@/1-542
P31636|SC5A4_PIG/1-661
sp|P11170|SC5A1_RABIT/1-663
sp|P13866|SC5A1_HUMAN/1-665
sp|P26429|SC5A1_PIG/1-606
sp|P53791|SC5A1_SHEEP/1-665
sp|P53790|SC5A1_RAT/1-666
sp|P26430|SC5A2_RABIT/1-673
sp|Q28610|SC5AA_RABIT/1-598
sp|Q28728|SC5AB_RABIT/1-675
sp|P31637|SC5A3_CANLF/1-719
M500
